# Amendments to the Constitution

**Published:** 1867-06

**Authors:** 


AMENDMENTS TO CONSTITUTION.

On motion of Dr. Cox, the President was directed to appoint a committee of five, to take into consideration such amendments and alterations in the plan of organization of the Association as may tend to remedy existing defects, and contribute to its efficiency—to report in 1868.

